# Development of recombinase polymerase amplification assays for rapid and visual detection of canine distemper virus infecting giant panda

**DOI:** 10.1186/s12917-021-02880-3

**Published:** 2021-04-23

**Authors:** Pei Huang, Yue Yu, Xianyong Meng, Tiecheng Wang, Feihu Yan, Entao Li, Zhikang Shi, Hongbin He, Songtao Yang, Xianzhu Xia, Jianzhong Wang, Na Feng

**Affiliations:** 1 College of Veterinary Medicine, Jilin Agricultural University, Changchun, China; 2 Key Laboratory of Jilin Province for Zoonosis Prevention and Control, Institute of Military Veterinary, Academy of Military Medical Sciences, Changchun, China; 3 College of Life Sciences, Shandong Normal University, Jinan, China

**Keywords:** Canine distemper virus, Giant panda, Reverse transcription recombinase polymerase amplification, Nucleic acid visualization assay

## Abstract

**Background:**

Canine distemper virus (CDV) is an enveloped negative-strand RNA virus that exhibits a high mutation rate and continuously expands the range of hosts. Notably, CDV has infected giant panda with spill over from viral reservoirs in canines. Giant pandas (*Ailuropoda melanoleuca*), especially captive pandas, are known to be susceptible to natural infection with CDV. The high fatality rate of CDV poses a serious threat to the safety of the giant panda population. However, vaccines or drugs for canine distemper in giant pandas have not been developed to date. Therefore, a rapid test that can achieve accurate onsite detection of CDV is important to enable the timely implementation of control measures. In this study, we established a nucleic acid visualization assay for targeting the CDV N gene by using combines reverse transcription recombinase polymerase amplification with a closed vertical flow visualization strip (RT-RPA-VF).

**Results:**

The RT-RPA-VF assay does not require sophisticated equipment, and it was determined to provide rapid detection at 35 °C for 30 min, while the limit of detection was 5 × 10^1^ copies/μl RNA transcripts and 10^0.5^ TCID_50_ ml^− 1^ viruses. The results showed that the assay was high specific to CDV and had no cross-reactivity with other viruses infecting the giant panda. Compared with RT-qPCR, RT-RPA-VF assay had a sensitivity of 100% and a specificity of 100% in 29 clinical samples. The coincidence rate between RT-RPA-VF and RT-qPCR was 100% (kappa = 1), indicating that the RT-RPA-VF assay possessed good diagnostic performance on clinical samples.

**Conclusions:**

The RT-RPA-VF provides a novel alternative for the simple, sensitive, and specific identification of CDV and showed great potential for point of care diagnostics for captive and wild giant panda.

## Background

Canine distemper (CD) is a highly contagious systemic viral disease caused by canine distemper virus (CDV); this disease was initially described as an infectious disease of domestic dogs and has been known since 1760 [1]. Viral transmission occurs via aerosols or by direct contact of susceptible animals with the various fresh body secretions of infected animals [2]. In recent years, spillovers of a virulent CDV strain constantly outbreak in dogs and wildlife animals, and the host range of CDV is expanding, including *Canidae, Procyonidae, Ailuridae, Ursidae, Mustelidae, Felidae, Hyaenidae,* and *Phocidae* [3].

The giant panda (*Ailuropoda melanoleuca*), which belongs to the order Carnivora, is a flagship animal one of the oldest existent species; the giant panda is not only known as China’s treasure but is also called the living fossil. In addition, the giant panda is listed as vulnerable by the International Union for Conservation of Nature [4], with a total wild population size of approximately 2000 [5]. Among all factors threatening the current global population of endangered animals, CDV is one of the leading infectious disease killer, posing a serious long-term threat [6, 7]. It was reported as early as 1997 that CDV caused the deaths of captive giant pandas when three pandas died at Chongqing Zoo [6]. Most recently, we have reported CDV outbreaks in the giant panda with a morbidity of 27% and mortality of 23% in the Shaanxi Rare Wild Animal Rescue and Breeding Research Center, China, between December 2014 and April 2015 [8].

Since there is no CDV vaccine specific for the giant panda, in addition to veterinary care and management, it is necessary to detect a CDV infection as early as possible to initiate the appropriate control measures and prevent further spread among populations of the giant panda. The methods that have been established to detect giant panda CDV, similar to the current diagnostic methods used for CDV in dogs, primarily include virus isolation and the detection of viral RNA by conventional, nested and real-time RT-PCR [9–11]. However, these methods require relatively sophisticated equipment with experienced technicians and are time-consuming, since it may take several days or even weeks to transport the field samples to a well-equipped specialized central laboratory for diagnosis. So, these assays are unsuitable for use in underequipped laboratories and in the field. Accordingly, a portable, simple, rapid nucleic acid detection test that is as accurate as PCR is needed to detect the virus at the sites where infections occur.

Recombinase polymerase amplification (RPA) is a novel isothermal amplification technology that was created in 2006. This method simulates the amplification process of bacteriophage gene replication under the action of three kinds of enzymes (recombinant enzymes, single-stranded binding proteins, and strand-displacing DNA polymerase) [12]. RPA technology is not only easy to operate but also has low requirements for equipment. This technology can complete the exponential amplification target sequence within 30 min at a relatively low temperature. Meanwhile, RPA has high specificity and sensitivity. These properties of RPA are more suitable for low-resource settings. To the best of our knowledge, the use of a reverse transcription RPA as a diagnostic tool for CDV in giant pandas has not been reported previously. Our study aimed to establish sensitive, rapid, and portable nucleic acid visualization assays against CDV, composed of reverse transcription RPA and a closed vertical flow visualization strip (RT-RPA-VF). It is important to effectively and rapidly diagnose CDV in giant pandas to provide timely treatment and prevent the spread of disease.

## Results

### Optimizing the RT-RPA-VF reaction conditions

To improve sensitivity, the RNA transcripts were employed as templates for optimizing the reaction conditions of the RT-RPA-VF assay for CDV, including temperatures and times.

First, various synthesized RNA transcripts were utilized as templates in the RT-RPA-VF assay. Then, all reactions were individually performed at different temperatures (44 °C, 42 °C, 39 °C, 37 °C, 35 °C, and 33 °C) for 20 min. Table 1 shows that this assay had a wide reaction temperature range, but all three replicates were positive at 35 °C. Therefore, 35 °C was the optimum temperature for the RT-RPA-VF assay against CDV.

**Table 1:** Reaction temperature optimization for RT-RPA-VF assays targeting the CDV N gene

Temperature	No. positive/ no. replications								
	Synthesized RNA transcript dilution (5 × copies/μl)								
	10^8^	10^7^	10^6^	10^5^	10^4^	10^3^	10^2^	10^1^	N
44 °C	3/3	3/3	3/3	3/3	3/3	0/3	0/3	0/3	0/3
42 °C	3/3	3/3	3/3	3/3	3/3	0/3	0/3	0/3	0/3
39 °C	3/3	3/3	3/3	3/3	3/3	1/3	0/3	0/3	0/3
37 °C	3/3	3/3	3/3	3/3	3/3	3/3	2/3	0/3	0/3
35 °C	3/3	3/3	3/3	3/3	3/3	3/3	3/3	0/3	0/3
33 °C	3/3	3/3	3/3	3/3	3/3	3/3	1/3	0/3	0/3

At the optimal reaction temperatures described above, the different times (15 min, 20 min, 25 min, 30 min, and 35 min) were screened. According to the highest dilution at which all three replicates were positive, the optimal reaction time was 30 min, as shown in Table 2.

**Table 2:** Reaction time for RT-RPA-VF assays targeting the CDV N gene

Time	No. positive/ no. replications								
	Synthesized RNA transcript dilution (5 × copies/μl)								
	10^7^	10^6^	10^5^	10^4^	10^3^	10^2^	10^1^	10^0^	N
15 min	3/3	3/3	3/3	3/3	0/3	0/3	0/3	0/3	0/3
20 min	3/3	3/3	3/3	3/3	3/3	3/3	0/3	0/3	0/3
25 min	3/3	3/3	3/3	3/3	3/3	3/3	1/3	0/3	0/3
30 min	3/3	3/3	3/3	3/3	3/3	3/3	3/3	0/3	0/3
35 min	3/3	3/3	3/3	3/3	3/3	3/3	3/3	0/3	0/3

### Sensitivity and specificity

To evaluate the sensitivity of the RT-RPA-VF assay for CDV, the tenfold RNA transcripts ranging from 5 × 10^6^ to 5 × 10^0^ copies/μl were detected. As the result indicate that red band on the test line was still visible during in 5 × 10^1^ copies/μl RNA transcripts, the limit of detection for the RT-RPA-VF assay was determined to be 5 × 10^1^ copies/μl RNA transcripts.

**Fig. 1:**
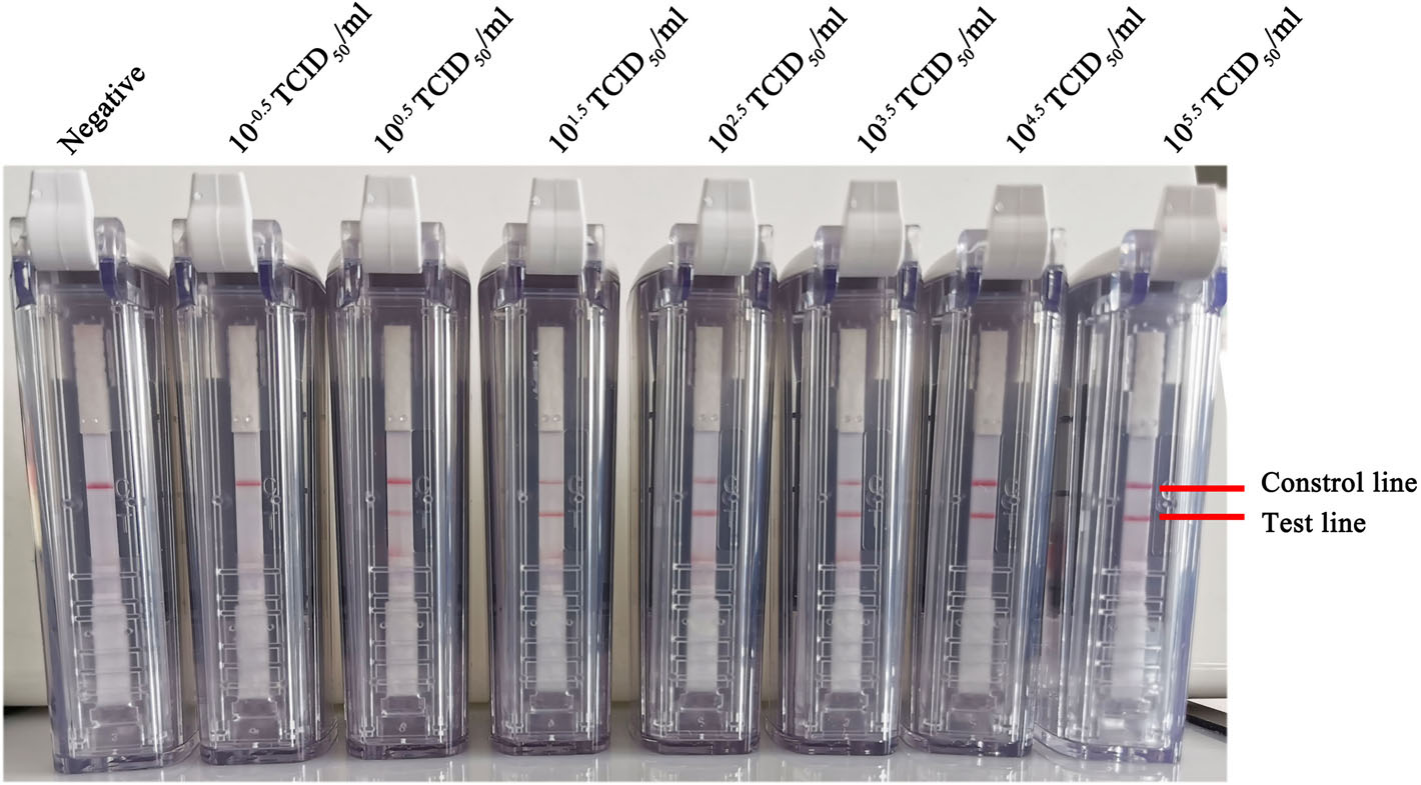
RT-RPA-VF assay limits of detection with CDV RNA extracted from strains that were serially diluted tenfold

The CDV-infected cells were serially diluted tenfold, then total RNA from 200 μl various titers (10^5.5^ TCID_50_ ml^− 1^ - 10^-0.5^ TCID_50_ ml^− 1^) were individually extracted to analyze the sensitivity of RT-RPA-VF assay. Negative results were observed when the concentration was lower than 10^0.5^ TCID_50_ ml^− 1^ virus in Fig. 1. The above data confirm that this assay could detect at least 10^0.5^ TCID_50_ ml^− 1^ virus.

**Fig. 2:**
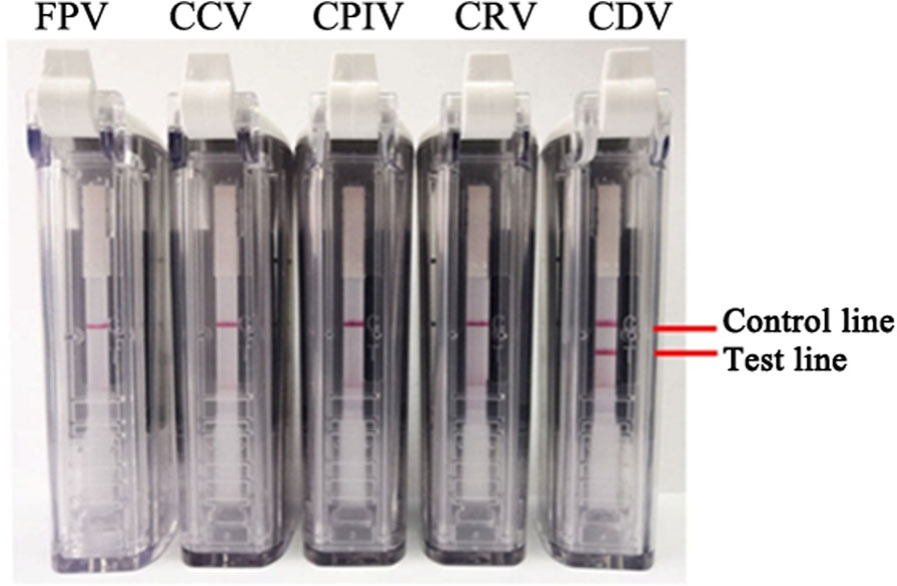
The specificity of the RT-RPA-VF assays against the CDV N gene

According to previous reports, clinical symptoms similar to CD can also be caused by FPV, CCV, CPIV and CRV; therefore, identification of the virus is difficult to achieve by clinical symptoms alone. To evaluate the specificity of the RT-RPA-VF assay for CDV, the nucleic acids from CDV, FPV, CCV, CPIV and CRV were detected by RT-RPA-VF assay. Given that a nonspecific reaction wasn’t observed in the no-target gene, only CDV RNA could be amplified by RT-RPA-VF assay, as shown in Fig. 2. Thus, this assay was determined not to cause cross-reactions with FPV, CCV, CPIV and CRV.

### Detection of clinical samples by RT-PCR, RT-qPCR and RT-RPA-VF assays

Twenty-nine field samples from giant pandas previously reported were diagnosed using the RT-RPA-VF assay [8]. Table 3 shows that 10 of 10 tissue samples, 2 of 4 oral swabs, 1 of 1 urine sample, 1 of 5 fecal samples and 1 of 1 blood sample were all positive by RT-PCR, RT-qPCR and RT-RPA-VF assays. The only difference was the test results of nasal swabs, 4 of 8 nasal swabs were positive via the RT-PCR, however, 5 of 8 nasal swabs were positive using the RT-qPCR and RT-RPA-VF assay (Table 3), the mean cycle threshold value (Ct) in RT-qPCR testing of the nasal swab that was false-negative by RT-PCR was 37.13. In summary, the coincidence rate between the RT-RPA-VF and RT-qPCR assay against CDV was 100% (kappa = 1), higher than that of RT-PCR with 96.5% (kappa = 0.922). RT-RPA-VF assay had a sensitivity of 100% and a specificity of 100% in 29 clinical samples.

**Table 3:** RT-RPA-VF, RT-PCR and RT-qPCR assays identify CDV in various clinical samples

Samples	RT-RPA-VF		RT-PCR		RT- qPCR	
	Pos./total	Neg./total	Pos./total	Neg./total	Pos./total	Neg./total
Tissue swabs	10/10	0/10	10/10	0/10	10/10	0/10
Nasal swabs	5/8	3/8	4/8	4/8	5/8	3/8
Oral swabs	2/4	2/4	2/4	2/4	2/4	2/4
Stool	1/5	4/5	1/5	4/5	1/5	4/5
Urine	1/1	0/1	1/1	0/1	1/1	0/1
Blood	1/1	0/1	1/1	0/1	1/1	0/1

## Discussion

In recent years, to avoid reliance on sophisticated thermal instruments of conventional molecular diagnostics for canine CDV, isothermal amplification assays have been developed. For example, Cho, et al. and Liu, et al. established a RT-LAMP assay for the detection and differentiation of canines infected with wild-type CDV from canines vaccinated with attenuated vaccine, respectively. The sensitivity of RT-LAMP was as low as 10^− 1^ TCID_50_ ml^− 1^ [13, 14]. However, the amplified products are detected by agarose gel electrophoresis, which might be subjected to aerosol contamination due to opening of the reaction tube, leading to false-positive results. In addition, at least four primers are required for this assay (two internal and two external), and six binding sites are required, which limits the ease and flexibility of oligonucleotide design. In addition, the amplifying process requires approximately 60 min, and results can be usually observed visually after 30–60 min [15].

Another isothermal amplification technique applied widely to detect pathogens is RT-RPA. Previously, Wang, et al. developed a real-time RT-RPA assay for canine CDV, which can detect 31.8 copies per reaction of RNA transcripts in 12 min [16], but this assay is only employed in advanced laboratories because it requires sophisticated real-time PCR instruments. Subsequently, Wang, et al. adjusted their design and developed a RT-RPA assay combined with lateral flow strips (LFS RT-RPA), which still had a high sensitivity of 94 copies RNA visible to the naked eye on the lateral flow strip [17]. The primers and probe sequences of LFS RT-RPA designed by Wang et al. are containing 13 mismatch bases compared with giant panda/SX/2014 isolated from giant panda both belong to Asia-1 genotype. According to previous research reports, 9 mismatches between RPA primer pairs and probes will affect the efficiency of RPA amplification or even inhibit amplification. So in this study, we developed a rapid, sensitive and simple method for detection of CDV in giant pandas based on the RT-RPA assay combined with a closed vertical flow visualization strip with primers and an nfo probe targeting the N gene. Our data showed that the limit of detection of the RT-RPA-VF assay was 5 × 10^1^ copies/μl RNA transcript and 10^0.5^ TCID_50_ ml^− 1^ for CDV, respectively, which was roughly consistent with the sensitivity of the LFS RT-RPA (94 copies RNA transcript).

RPA tolerates a wide range of reaction temperatures and does not require the reaction temperature to be precisely controlled. Previous studies have shown that RPA retains reliable functionality between 31 °C and 43 °C [18] and even between 30 °C and 45 °C [19, 20], although the standard TwistAmp™ kits are configured to operate optimally in the temperature range of 37 °C to 42 °C. Our data showed that between 33 °C and 37 °C, the RT-RPA-VF assay performed very well, with as few as 5 × 10^2^ copies/μl CDV RNA transcripts testing positive under the incubation time of 20 min. Incubation temperatures of 35 °C provided optimal conditions in which all 3 replicates were positive (Table 1). When incubation times were extended to more than 25 min, positive RPA signals could be seen at a lower template of 5 × 10^1^ copies/μl, even at more than 30 min, resulting in all 3 replicates being positive (Table 2). Thus, the optimal temperature and time were determined to be 35 °C and 30 min, respectively.

**Fig. 3:**
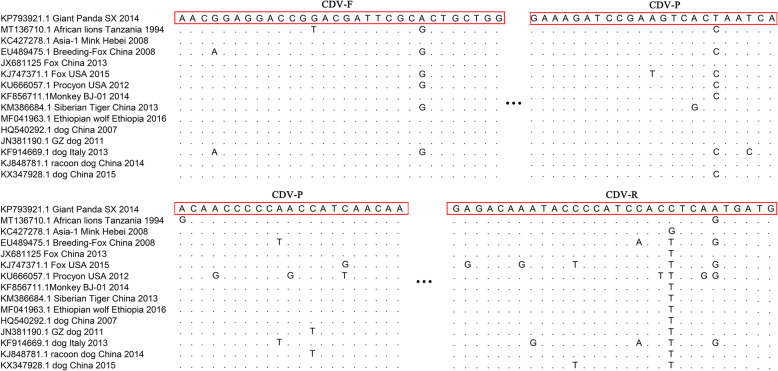
Primers and probe specificity of the RT-RPA-VF assays for the CDV N gene

The sensitive, specific, simple, and rapid diagnostic RT-RPA-VF assay employed giant panda/SX/2014 as the diagnosis object was successfully established. It could be applied to the early rapid detection of CDV infecting giant pandas. CDV strains circulating among canid and non-canid species worldwide were classified into 17 lineages: America-1 (vaccine strains), America-2, America-3, America-4, America-5 Arctic-like, Rockborn-like, Asia-1, Asia-2, Asia-3, Asia-4, Africa-1, Africa-2, European Wildlife, Europe/South America-1, South America-2 and South America-3 [21]. It was reported that RPA is tolerant to 5–9 mismatches in primers and probe, showing no influence on the performance of the assay [22, 23]. There were only 1–9 mismatches between the primers and probe employed in this study and the sequences of other strains (Fig. 3). Hence, it may have the potential to be utilized for the detection of various genotypes, on account of that the RT-RPA-VF assay targeted the conserved N gene of CDV.

Because early, rapid and accurate epidemiological surveillance of infected giant pandas with CDV is important to isolate the animals and adopt an appropriate treatment, we further evaluated RT-RPA-VF assay using clinical giant panda samples. Compared with RT-qPCR, RT-RPA-VF assay for CDV N gene had a sensitivity of 100% and a specificity of 100% in 29 clinical samples. No discrepancy was found in clinical samples even containing low levels of viral load (Ct = 37.13, RT-qPCR). The coincidence rate between the RT-RPA-VF assay against CDV and RT-qPCR was 100% (kappa = 1). But one nasal swab with a Ct value of 37.13 in RT-qPCR, was observed false-negative in the RT-PCR testing, it may be related to the lower sensitivity of RT-PCR compare with RT-qPCR, RT-RPA assays [23, 24].

Depending on MLV added to into the TwistAmp™ nfo reaction system, viral RNA was employed as the template directly for the RT-RPA-VF assay without an additional step of reverse transcription to cDNA, which consumes approximately 30–60 min [24, 25]. RNA purification was performed by using a commercial RNA extraction kit, which is a limitation of this study. Magnetic bead-based extraction methods will be used in future research to prepare the RPA templates from clinical samples. Extraction using magnetic beads is easy and time-saving, and it does not require potentially dangerous procedures or specialized laboratory equipment [26, 27]. The combination of the RT-RPA-VF assay with magnetic bead-based nucleic acid extraction techniques represents a very promising point-of-care detection method.

## Conclusions

In summary, RT-RPA-VF assay against CDV was simple, sensitive, and specific, this assay can detect as little as 5 × 10^1^ copies/μl RNA transcript and 10^0.5^ TCID_50_ ml^− 1^ for CDV within 35 min, and no sophisticated equipment was needed; thus, this assay can be applied for detection and active surveillance in low-resource areas. If this assay is combined with virus inactivation and simplified RNA extraction, it can be use in point-of-care testing for captive and wild giant panda.

## Methods

### Rationale of RT-RPA-VF

**Fig. 4:**
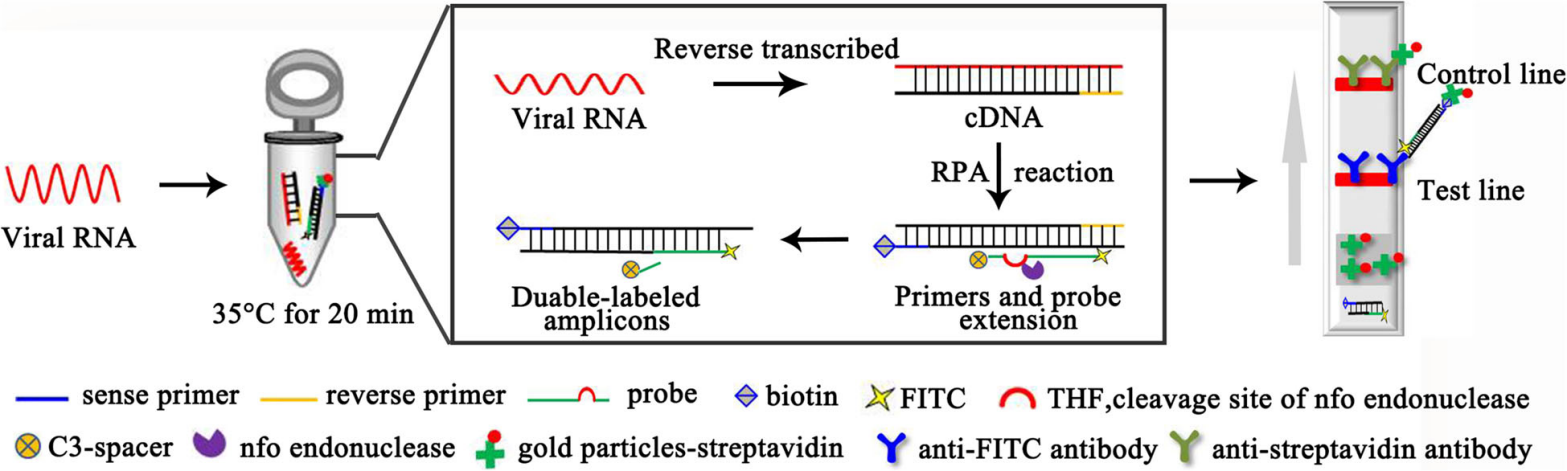
Schematic illustration of RT-RPT-VF assay targeting the CDV N genes. The RT-RPA was performed at a constant temperature. Firstly, Viral RNA is transcribed into cDNA by transcriptase. Secondly, the recombinase, strand-displacing DNA polymerase, SSB (not shown) and primers initiated amplification reaction. Another enzyme, nfo can cleave THF site when the probe hybridizes to its target sequence, result in the departure of C3-spacer and probe extension. The tube was placed in a closed device of vertical flow visualization strip device for detecting the RT-RPA products

The principle of the RT-RPA reaction has been described in detail in a previous report [12]. As shown in Fig. 4, RNA was first reverse transcribed to cDNA using reverse primer and MLV reverse transcriptase. Following, primers and the probe triggered extension events by recombinant enzymes, single-stranded binding proteins (SSB), and strand-displacing DNA polymerase. To combine a closed vertical flow visualization strip, the sense primer was labeled with biotin at the 5′ end, the probe was modified, including the 5’ end-labeled FITC, the tetrahydrofuran (THF) residue replaced a nucleotide, and the 3’ end blocked the C3 spacer. Notably, THF is a recognition and cleavage site of nuclease–endonuclease IV (nfo). When the probe hybridizes a single DNA strand, nfo cleaves the probe at the THF position and removes the blocking group, thereby extending the probe under the action of polymerase. This design improves the specificity of the RT-RPA-VF assay. Finally, the amplicons are indirectly labeled with FITC and biotin and can be captured by an anti-FITC antibody and gold particles-streptavidin that is fixed on a closed disposable vertical flow visualization strip, and aggregated gold particles are presented as a red band on the test line [28].

### Virus strains and clinical samples

The cell culture lysates were collected after being infected for 30 h with the giant panda/SX/2014 strain of CDV (GenBank accession no. KP793921). Feline parvovirus (FPV**,** GenBank accession no. KX900570), canine coronavirus (CCoV, GenBank accession no. AY390344.1), canine parainfluenza virus (CPIV, GenBank accession no. KY114804.1), and canine rotavirus (CRV, GenBank accession no. FJ669132.1) were stored in our laboratory. All virus strains were stored at − 80 °C.

Ten tissue samples, seven nasopharyngeal swabs, one stool sample, one urine sample and one blood sample from five giant pandas that died of CDV infection were provided by Shaanxi Rare Wild Animal Rescue and Research Center [8]. Five nasopharyngeal swabs and four stool samples were collected from giant pandas of the China Conservation and Research Center for the healthy giant panda. All positive clinical samples were detected by RT-PCR and the product was further sequenced to validate. Phylogenetic analysis and multiple sequence alignments based on the H gene sequence revealed that all of them belong to the Asia-1 cluster [8]. All clinical samples were stored at − 80 °C.

### Preparation of templates

DNA/RNA was individually purified from various cell culture lysates infected with the viruses mentioned above using the TIANamp Virus DNA/RNA Kit (TIANGEN Company, Beijing, China) according to the manufacturer’s instructions. All of the nucleic acids were eluted by 50 μl of DEPC water, and 2 μl was added to the reaction system.

The RNA transcripts of position 1260 to 1517 of the CDV N gene sequence (giant panda/SX/2014 strain) was synthesized and purified by Bao Biological Co., Ltd. (Dalian, China) at a concentration of 5 × 10^11^ copies/μl and stored at − 80 °C.

### Primer and probe design of RT-RPA-VF assay

Fifteen strains of the CDV N gene sequence that had been published in GenBank were aligned by MEGA7 software. As shown in Fig. 3, a 158-nt relatively conserved sequence was screened as a target to design primers and probe. All of the primers and probe were synthesized by Shanghai Biological Co., Ltd. (Shanghai, China). The sense primer CDV-F (5′-Biotin-AACGGAGGACCGGACGATTCGCACTGCTGG-3’), reverse primer CDV-R (5’-CATCATTGAAGTGGATGGGGTATTTGTCTC-3’), and probe CDV-P (5′-FITC-CTCTTGTTGATGGTTGGGGGTTGTTGATTGGT[THF]GACTTCGGATCTTTC-C3 Spacer-3’) were designed to target 158-bp sections of the N gene. The sense primer was labeled with biotin at the 5’ end, the probe was modified, including the 5′ end labeled FITC, the tetrahydrofuran (THF) residue replaced a nucleotide, and the 3′ end blocked the C3 spacer (Table 4).

**Table 4:** Primers and probe information

Assay	Primers name	Position	Target gene	Sequence (5′ – 3′)
RT-RPA-VF	CDV-F	1310–1339	N	Biotin-AACGGAGGACCGGACGATTCGCACTGCTGG
	CDV-R	1438–1467		CATCATTGAAGTGGATGGGGTATTTGTCTC
	CDV-P	1374–1420		FITC-CTCTTGTTGATGGTTGGGGGTTGTTGA TTAG[THF]GACTTCGGATCTTTC-C3 Spacer
RT-PCR	GBJD-F	7823–7842	H	CGAGTCTTTGAGATAGGGTT
	GBJD-R	8258–8277		CCTCCAAAGGGTTCCCATGA
RT-qPCR	N-F	905–931		AGCTAGTTTCATCTTAACTATCAAATT
	N-R	965–987	N	TTAAATCCCCAGAAAACTCATGC
	N-P	934–963		FAM-ACCCGAGAGCCGGATACATAGTTTCAATGC-BHQ

All of primers and probe positions were based on the giant panda/SX/2014 strain of CDV (GenBank accession no. KP793921.1)

### RT-RPA-VF assay reaction conditions

The RT-RPA-VF assay was performed using TwistAmp nfo reagents (TwistDx Ltd., Cambridge, United Kingdom). The reaction was prepared in a 50 μl volume including 29.5 μl of rehydration buffer, 2.1 μl CDV-F/R primers (10 μmol/l), 0.6 μl probe CDV-P (10 μmol/l), 2 μl of RNA template, 2 U MLV reverse transcriptase (Invitrogen, Shanghai, China), then filling up to 50 μl with DEPC water. The premixed solution was transferred to a reaction tube with freeze-dried powder containing three enzymes necessary for RPA amplification. Then, 2.5 μl of 280 mM MgCl_2_ was added to the tube cap. After mixing and instant centrifugation, the tubes were placed in a constant temperature metal bath at 35 °C for 20 min. Finally, this reaction tube was inserted into a closed vertical flow visualization strip (Ustar Biotech Co., Ltd., Hangzhou, China) to read the detection result. All of the RT-RPA reactions were repeated three times. A positive result could be observed as two red bands on the strip, differ from only a red band in the control line with the negative result.

### Analytical sensitivity and specificity

Tenfold serial dilution of CDV RNA transcripts (ranging from 5 × 10^6^ to 5 × 10^0^ copies/μl) were employed as templates for the analytical sensitivity of the RT-RPA-VF assay. For evaluation of the detection limits, the RNAs of the giant panda/SX/2014 strain of CDV ranging from 10^5.5^ to 10^–0.5^ TCID_50_ ml^− 1^ were subjected to the RT-RPA-VF assay.

The specificity of the RT-RPA-VF assay was evaluated with FPV, CCV, CPIV and CRV, which can cause respiratory or digestive symptoms similar to CDV. The RNAs from FPV, CCV, CPIV and CRV were purified and detected by the RT-RPA-VF assay against CDV.

### RT-PCR for canine distemper virus

According to the China’s diagnostic protocols for CDV, the primers of RT-PCR against N gene were synthesized by Shanghai Biological, Co., Ltd. The primers information was further described in Table 4. Extracted RNA from sample was tested by RT-PCR. The 50 μl volume of first reaction was prepared included 10 μl of 5 × AMV buffer, 1 μl of Oligo (dT) 18primer, 1 μl of Random Primer pd. (N)9, 5 μl of 10 mM dNTP, 4 μl of Reverse Transcriptase AMV, 1 μl of Recombinant RNase Inhibitor, 28 μl of RNA was performed at 42 °C for 60 min. All reagents were purchased from Bao Biological, Co., Ltd. (Dalian, China).

Following, the 25 μl volume of the second reaction included 2 μl of cDNA, 5 μl of 5 × FastPfu Buffer, 1 μl of 10 μM primers, 2.5 μl of 2.5 mM dNTPs, 0.5 μl of FastPfu, 13 μl of ddH_2_O, was incubated at 94 °C for 3 min, followed by 35 cycles at 95 °C for 20 s, 52 °C for 20 s, and 72 °C for 30 s with a final extension at 72 °C for 5 min. The final PCR products were read by electrophoresis on a 1% agarose gel. All reagents were purchased from TransGen Biotech, Co., Ltd. (Beijing, China).

### RT-qPCR for canine distemper virus

RT-qPCR assay for CDV was performed using previously reported methods on clinical samples [10]. The primers and probe were synthesized by Bao Biological, Co., Ltd. (Dalian, China). Detailed information of primers and probe was shown in Table 4. According to the manufacturer’s instructions for One Step PrimeScript™ RT-PCR Kit (Bao Biological, Co., Ltd., Dalian, China), a premixed solution was prepared containing 12.5 μl of 2 X One Step RT-PCR BufferIII, 0.5 μl of TaKaRa Ex Taq HS (5 U/μl), 0.5 μl of PrimeScript RT Enzyme Mix II, 7.5 μl DEPC water, 0.5 μl of 10 μM primers, 1 μl of 10 μM probe, and 2 μl of template.

The amplification reaction was performed on an Bio-Rad CXF96 real-time PCR instrument (Bio-Rad Company, California, USA), and thermal cycling was performed at 42 °C for 5 min, followed by 94 °C for 10 s and 40 cycles of 94 °C for 5 s and 60 °C for 30 s. Each group included a positive and negative control. A mean threshold cycle (CT) values were measured in triplicate.

## Data Availability

The datasets generated and analysed during the current study are available in the National Center for Biotechnology Information (NCBI) repository, under these GenBank accession numbers KP793921.1, MT136710.1, KC427278.1, EU489475.1, JX681125, KJ747371.1, KU666057.1, KF856711.1, KM386684.1, MF041963.1, HQ540292.1, JN381190.1, KF914669.1, KJ848781.1, KX347928.1, KX900570, AY390344.1, KY114804.1, FJ669132.1.

## Abbreviations

CDV: Canine distemper virus
RT-RPA-VF: Transcription recombinase polymerase amplification with a closed vertical flow visualization strip
RNA: Ribonucleic acid
TCID_50_: Median tissue culture infective dose
RT-PCR: Reverse transcription-polymerase chain reaction
RT-qPCR: Real-time reverse transcription-polymerase chain reaction
FPV: Feline parvovirus
CCV: Canine coronavirus
CPIV: Canine Parainfluenza virus
CRV: Canine rotavirus
cDNA: Complementary deoxyribonucleic acid
FITC: Fluorescein isothiocyanate
THF: Tetrahydrofuran
nfo: Nuclease–endonuclease IV
LFS: Lateral flow strip
CT: Threshold cycle
